# The Impact of Enforced Working from Home on Employee Job Satisfaction during COVID-19: An Event System Perspective

**DOI:** 10.3390/ijerph182413207

**Published:** 2021-12-15

**Authors:** Jun Yu, Yihong Wu

**Affiliations:** School of Economics & Management, Shanghai Maritime University, Shanghai 201306, China; yihongwu@stu.shmtu.edu.cn

**Keywords:** COVID-19 pandemic, working from home, job satisfaction, event system theory, job characteristic, fsQCA

## Abstract

During the COVID-19 pandemic, working from home (WFH) became the only option for many organizations, generating increasing interest in how such arrangements impact employee job satisfaction. Adopting an event system perspective, this study employed an online survey to capture the WFH experiences of 256 workers from 66 Chinese enterprises during the pandemic. Using fuzzy-set qualitative comparative analysis (fsQCA), the study examined how satisfaction was affected by five job characteristics when working from home: longevity (time), home workspace suitability (space), job autonomy (criticality), digital social support (novelty) and monitoring mechanisms (disruption). The findings reveal that three configurations promote employee job satisfaction and that a suitable home workspace is a core condition. In the absence of a suitable workspace, digital social support and an appropriate monitoring mechanism, long-term WFH was found to undermine job satisfaction. However, job autonomy is not a necessary condition for employee job satisfaction. These findings have clear implications for theory and practice.

## 1. Introduction

As a consequence of the COVID-19 pandemic, about 72% of employees worldwide were required to switch overnight to working from home (WFH) [1]. According to a Survey Monkey report, more than 89% of employees surveyed (*n* = 9059) were satisfied with their WFH arrangements [2]. However, a Martec Group 2020 study reported that only 32% of respondents (*n* = 1214) were satisfied with their WFH arrangements during the COVID-19 pandemic [3]. Similarly, a survey conducted by the Institute for Employment Studies found that 50% of respondents (*n* = 500) were dissatisfied with their current WFH arrangements; of those, 46% attributed their dissatisfaction to irregular working hours, while 33% cited loneliness and 21% expressed concerns about job security [4]. It seems important, then, to understand why job satisfaction experiences differ among the employees who engaged in WFH during the COVID-19 pandemic and how WFH might be designed to improve those experiences.

As a work practice, WFH means that an employee performs work-related activities from their home rather than being physically present at an employer location, typically using digital technology [5]. Previous findings regarding the relationship between WFH and employee job satisfaction are inconclusive [6]. WFH falls into the category of remote work; existing research has identified factors that increase employee job satisfaction of remote work, including income, working hours, free time, appropriate physical activity [7], the frequency of remote work [8], work location [9], social interaction and technical support [10], position, company training, relationship with supervisors and environmental conditions at work [11]. Beyond that, scholars initially investigated the associations between WFH and employee job satisfaction in terms of individual needs. According to signalling theory, observable organizational actions can be interpreted as a signal of unobservable characteristics, such as the organization’s concern for employee welfare. After receiving the signal, employees tend to adopt a more positive attitude [12]. Research based on this theory proposes that WFH is traditionally presented as an employee benefit that contributes to a positive work attitude [13] and is usually seen as a work–family enrichment measure [14]. Role balance theory suggests that individuals who can successfully balance multiple roles (employee, spouse, etc.) will experience more positive effects than those who achieve less balance [15]. According to these studies, WFH enhances job satisfaction by contributing to work–family life balance [16]. On the other hand, self-determination theory emphasizes how WFH fulfils personal psychological needs (e.g., autonomy, competence, relatedness) as a driver of job satisfaction [17]. Scholars have subsequently noted that individual and job characteristics can moderate the relationship between WFH and job satisfaction. Both social exchange theory and organizational justice theory posit that people seek a balance between an investment in a relationship and what they receive in return [18,19]. Studies based on social exchange theory contend that social support in the workplace can strengthen home-based workers’ job satisfaction and sense of embeddedness [20]. According to justice theory, employees who are unable to participate in WFH because of technical or management issues tend to compare their situation with that of home office workers, perceive inequity and unfairness, and attempt to remove such feelings by reducing their job satisfaction and intention to stay [21]. The essence of job characteristic theory is that certain job characteristics may increase the probability that individuals will find their work meaningful, take responsibility for work outcomes, and have trustworthy knowledge of the results of their work, which can motivate task completion and enhance job satisfaction [22]. On that basis, job demands–resources theory divides job characteristics into demands and resources [23]. Studies based on these theories identify a suitable home workspace [24] and job autonomy [25] as likely sources of high-level job satisfaction. Role theory notes that individuals play multiple different roles in daily life that make different demands on time and energy commitments. These roles are often incompatible, which may lead to “inter-role conflict” [26], and WFH mitigates work–family conflict by reducing “inter-role conflict”, therefore increasing job satisfaction [27].

However, WFH also poses certain risks [28]. Boundary theory emphasizes the boundary between an individual’s work and non-work domains and the transition between various roles. The degree of segmentation or integration between employees’ work and non-work domains determines the success or failure of their role transitions [29]. Research informed by this perspective suggests that WFH can blur work–family boundaries and exacerbate work–family conflict if employees are unable to avoid working overtime or work-related disruption during breaks, which has been found to affect job satisfaction insignificantly [30] or negatively [31]. According to organizational support theory, employees tend to evaluate their performance more positively if the organization meets their social-emotional needs, rewards their work achievements and helps them in times of need [32]. Nonetheless, social isolation when WFH can undermine relationships with colleagues, resulting in job dissatisfaction [33]. The relationship between WFH and job satisfaction is also thought to be moderated by extent [34] or longevity [35] and by individual personalities and preferences [36,37].

These equivocal findings can be attributed to three deficiencies in existing studies on the relationship between WFH and employee job satisfaction. First, studies conducted prior to the pandemic focused mainly on voluntary home-based workers and sought to determine the type of employees who were suitable for working from a home office [38]. However, the enforcement of WFH during the COVID-19 pandemic deprived employees of choice [39]. For enterprises, existing management initiatives might not be sufficient to help employees cope with the extra pressure [40] due to inadequate operating conditions and organizational support for WFH implementation [41]. The existing literature cannot fully explain the impacts of enforced WFH during the COVID-19 pandemic [39], and this matters because organizations need to redesign and optimize WFH arrangements, taking account of how the job characteristics associated with enforced WFH affect job satisfaction.

A second deficiency is that previous studies typically explored the net effect of WFH on job satisfaction by treating job characteristics as moderating or mediating variables [5]. However, the uniqueness and novelty of COVID-19 altered some characteristics of WFH [42], given that the effect on job satisfaction is the result of multiple interacting job characteristics rather than a single factor [30]. As job characteristics also vary with context [9], it seems useful to explore which characteristics of WFH are most important and how they can be configured to maximize utility by treating WFH as an event [6].

Finally, a majority of existing studies emphasize the issues of job autonomy and social isolation associated with WFH [28] but fail to examine monitoring mechanisms. Compared with employees who work in the office, home-based workers are less likely to be subject to organizational supervision and control [43]. While the use of various technologies to monitor home-based workers can reduce employee procrastination [38], surveillance tools can also undermine productivity if workers feel untrusted or have concerns about privacy and security, which in turn can have a devastating impact on job satisfaction [44]. Indeed, the extensive use of technology may lead to an “autonomy paradox”: the greater the autonomy offered by WFH and technology, the more employees are likely to feel controlled [45]. It follows that the effective monitoring of home-based workers must balance autonomy and control, and this is crucial for understanding any improvements in WFH job satisfaction during the COVID-19 pandemic.

On that basis, we formulated the following research question: *How should enterprises configure the different job characteristics of WFH to improve employee job satisfaction during the COVID-19 pandemic?* Based on event system theory (EST) [46], the present study analyses optimal configurations in terms of how the longevity of WFH (LWFH), home workspace suitability (HWSS), job autonomy (JA), digital social support (DSS) and monitoring mechanisms (MM) affect employee job satisfaction (EJS). To that end, we employed fuzzy-set qualitative comparative analysis (fsQCA) to analyse the experiences of home-based workers from 66 Chinese enterprises during the COVID-19 pandemic.

The study makes four main contributions. First, by analysing how key characteristics of enforced WFH can be configured to enhance EJS, the study extends the WFH literature beyond voluntary contexts and informs the design of future office models. Second, the study enriches the literature on WFH supervision strategies by introducing the concept of MM for EJS. Third, the study augments existing research on job design and EJS by employing fsQCA to explore configurations of WFH job characteristics as antecedents of job satisfaction. Finally, the study contributes to EST by situating it proactively in the context of enforced WFH.

## 2. Theoretical Background and Literature Review

### 2.1. Event System Theory

According to EST, event strength, time and space determine an event’s degree of influence on an entity. Event strength comprises *criticality* (the event’s importance), *novelty* (the extent to which an event differs from current and past events) and *disruption* (the extent to which the event obstructs or subverts routine activities). Temporal characteristics, which distinguish events from constant features of the work environment, include event duration, timing and changes in strength. Finally, event space refers to the specific location where an event originates and how its effects spread; spatial characteristics include event origin, spatial dispersion and spatial proximity [46].

Depending on their source, events are categorized as *reactive* (if entities are forced to accept their occurrence) or *proactive* (if entities actively create them) [46]. As strong environmental events are more likely to alter behaviours [47], EST is typically used to determine the impact of reactive events on organizational outcomes, such as team knowledge absorption [48], team leadership [49] and organizational evolution [50]. Research on the individual-level mostly focuses on the impact of the strength of the COVID-19 event on individual innovation behaviour [51], job search behaviour [52], public emotional response [47], employees’ sense of job insecurity [53] and vaccination intention [54], but there is still a lack of studies on EJS during the COVID-19 pandemic based on EST. Among the few studies applying EST to proactive events, Lu et al. [55] explored the impact of tourism development on urban economies, and Hu et al. [56] investigated which attributes of enterprise safety training programmes promote employee safety behaviours.

The premise of the present study is that, from an EST perspective, WFH can be regarded as a proactive event for several reasons. First, we can fully comprehend the impact of enforced WFH on employee attitudes only by taking account of the interactions between WFH job characteristics [30]. Second, WFH fully conforms to the definition of an event as described by EST: WFH (1) is external to employees, (2) has a clear beginning and end point (This study relates to China, where prevention and control has entered the normalization stage. Employees can continue to work in the office after lockdown restrictions are completely lifted, working from home again following a sporadic outbreak. In that sense, there is a clear beginning and end time for WFH during the pandemic, and WFH has become a discrete event), (3) involves the intersection of organization and employees and (4) commands employees’ attention. On that basis, it is justifiable to conceptualize enforced WFH as an event. More specifically, EST offers a systematic perspective for exploring the combined effects of the job characteristics of enforced WFH on EJS during the COVID-19 pandemic.

### 2.2. Literature Review

Job characteristics are the essential attributes inherent in a task or job performed by an employee [22]. The characteristics of WFH refer to the nature of the job during the period of working from home [38]. According to job demands–resources (JD-R) theory, job characteristics can be categorized as job demands or job resources [23]. Job demands are elements that can cause stress, including workload [57], working hours [58] and working conditions, such as noise and temperature [59]. Job resources are physical, psychological, social or organizational aspects of work that can support employees and help them to maintain well-being. These include the suitability of the home workspace [60,61], the availability of digital resources and the Internet [61], job autonomy, social support [62], supervisory coaching and performance feedback [63] and promotion opportunities [64].

Based on EST and JD-R theory, the present study proposes the concept of MM to characterize the disruptive component of pandemic-enforced WFH as an event alongside JA (criticality) and DSS (novelty) as other aspects of event strength, and considers LWFH and HWSS as temporal and spatial features of the event, respectively. The focus on these five job characteristics relates to the two knowledge gaps addressed here. 

(1) According to JD-R theory, job demands interact with job resources to predict EJS. Job demands can reduce satisfaction if excessive work demands and pressure undermine workers’ health. However, if job resources are sufficient to balance those demands, employees are likely to be satisfied with their job [64]. In this regard, LWFH during the pandemic reflects the evolutionary nature of WFH when it is enforced by the crisis [59] and employee perceptions of work duration as an aspect of workload. To that extent, LWFH can be understood as a key job demand during this period. Along with HWSS, JA, DSS and MM as physical, psychological, social and organizational job resources, these five elements are crucial for any enterprise effort to improve EJS. (2) Job characteristics can interact with each other [64], inviting a study of their configurations. For example, long-term WFH is likely to aggravate occupational isolation and limit employee access to social support [65], as well as create work pressure and an “autonomy paradox” [66]. Social support in the workplace can increase employee autonomy and mitigate the negative effects of stress on satisfaction [67].

In light of how the COVID-19 pandemic has fundamentally changed our ways of working, the present study focuses on the effects of enforced WFH on job satisfaction in terms of individual evaluations and feelings [14] rather than specific job aspects [34]. The sections that follow discuss the mechanisms that determine the impact of the five conditions on EJS.

#### 2.2.1. Longevity of Working from Home and Job Satisfaction

Crisis-induced WFH events are evolutionary in nature [59], and most of the relevant studies argue that the relationship between LWFH and EJS is non-linear [34]. Short-term WFH reflected organizational concerns about employee health during the COVID-19 pandemic [68], causing employees to feel more positive about their work [69]. However, the isolation associated with long-term WFH limits social contact within and outside work [37], which increases the risk of frustration among home-based workers and so undermines job satisfaction [34]. Surprisingly, Golden et al. [65] found that individuals with high-quality monitoring mechanisms and undergoing long-term WFH reported the highest job satisfaction.

#### 2.2.2. Home Workspace Suitability and Job Satisfaction

The suitability of home working conditions encompasses “physical” elements (e.g., dedicated workplace, essential IT tools) and “mental” conditions (e.g., freedom from distractions and noise) [59] which impact significantly on employee satisfaction [57]. According to self-determination theory, IT tools enable home-based workers to share information across time and space boundaries [70] and help to fulfil the psychological need for interpersonal interaction, thus helping to improve job satisfaction [71]. Work adjustment theory asserts that a separate home workspace ensures clear structural boundaries between work and home and maintains job satisfaction by controlling distractions, such as children and noise [59]. On the other hand, Bellmann et al. [30] found no association between blurred home–work boundaries and job satisfaction.

#### 2.2.3. Job Autonomy and Job Satisfaction 

Job autonomy is the permitted extent of independence and discretion when performing professional tasks [22], including time and scheduling, and this is a key determinant of job satisfaction [72]. A majority of existing studies explain the relationship between employee JA and EJS in terms of JD-R theory and the resource-based view. Autonomy is an important job resource because it enables employees (1) to coordinate their work time to suit their preferences and schedule their work to ensure personal productivity and (2) to self-organize their work tasks to cope more effectively with stressful job demands [25], ensuring greater job satisfaction [23]. However, an opposing view suggests that flexible working hours can create insecurities related to performance evaluation criteria and supervisor expectations, adding to working time and stress and reducing job satisfaction [73].

#### 2.2.4. Digital Social Support and Job Satisfaction

Social support refers to assistance or emotional support provided by communication with others, especially in stressful situations [74]. Because of the quarantine measures introduced by the Chinese government during the pandemic, almost everyone must rely on online platforms for digital social support both in and outside of work [75,76]. According to social support theory, DSS during work provides the necessary emotional and instrumental resources to mitigate work–family conflicts, therefore promoting job satisfaction [77]. Similarly, DSS outside of work improves job satisfaction by compensating employees for the lack of interpersonal interaction during working hours and by providing a release from work pressure [76]. However, others have argued that the low-quality communication afforded by digital technologies may undermine job satisfaction by amplifying information uncertainty [20].

#### 2.2.5. Monitoring Mechanism and Job Satisfaction

Monitoring and evaluating employees is an essential component of WFH arrangements [78]. Control theory suggests that managers may place more emphasis on output control to address the challenges of monitoring homeworkers’ behaviour [79]. *Output control* emphasizes target-related performance [80,81], and *behavioural control* emphasizes task scheduling, with frequent monitoring of employee compliance with regulations. Organizations commonly use these two control methods to guide and communicate with employees. By helping to relieve their stress and improve their adaptability, these methods can contribute to increased job satisfaction [81]. In contrast, social exchange theory advocates for clan control, which seeks to promote appropriate behaviours by committing employees and managers to shared beliefs and values [82]. As this sharing is based on regular interactions between employees and managers, clan control helps to build healthy relationships between superiors and subordinates, thereby enhancing employee satisfaction [83]. On the other hand, Piccoli et al. [84] reported that strict control of home-based workers may reduce the effectiveness of coordination and communication and does not ensure job satisfaction.

**Figure 1:**
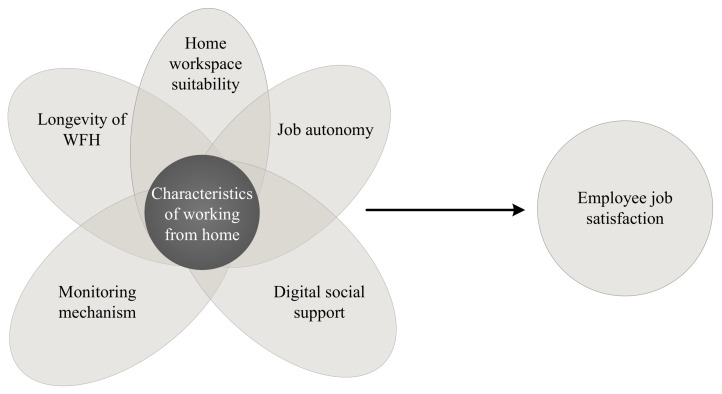
Research model.

In light of the intricate interactions among these five factors and the lack of research analysing the configurations of EJS, the present study can only review direct links between these factors and EJS, which are undoubtedly no more than a subset of all possible configurations. In addition, as existing studies of this relationship are inconsistent and contradictory, a new method of configuration analysis is needed to resolve these conflicts and explore unknown complementary sets. Figure 1 depicts the proposed research model.

## 3. Methodology

### 3.1. Sample and Procedure

To collect the data, we conducted an online questionnaire survey during the period June to September 2021. At the outset, each questionnaire was divided into two parts: Part A measured MM, and Part B measured LWFH, HWSS, JA, DSS and EJS, as well as control variables (Appendix A).

The sample was drawn from two sources. The main source comprised Executive Master of Business Administration (EMBA) students (who came from different provinces of China) at universities in Shanghai. With the help of Master of Business Administration education centres, 200 part-time EMBA students (who worked on weekdays as human resource (HR) managers) were randomly selected and informed by e-mail about the survey’s purpose. This group was chosen as the main target sample because they were likely to have a comprehensive knowledge of WFH and issues related to job satisfaction. For the other part of the sample, we chose 50 HR managers at random from the LinkedIn network of professional profiles, using *HR manager* as the filter criterion. We sent them a brief description of the study and an invitation to participate in the survey. It is worth noting that respondents were not known to the authors and were not drawn from the authors’ profiles. To improve the survey’s accuracy, we added the following filtering questions: (1) Did your enterprise practise enforced working from home during the COVID-19 pandemic? (2) Are you a HR manager? We excluded respondents whose company had not implemented enforced WFH or who were not HR managers. After confirming that the respondent qualified, we sent them the questionnaire, assuring them that their responses would remain anonymous and confidential. Of the HR managers who agreed to participate in the survey (*n* = 66), 52 were EMBA students, and 14 were LinkedIn users, mainly from manufacturing (31.5%), aerospace (21.2%), information technology (15.2%), internet services (13.9%), education (9.1%) and banking (7.6%). All respondents were asked to complete Part A of the questionnaire and e-mail Part A of the questionnaire directly to the authors after completion; they were then asked to send Part B of the questionnaire (with the company’s identifying code and the authors’ email addresses) to 4–6 employees who were working from home during the pandemic. After completing Part B of the questionnaire, employees e-mailed Part B directly to the authors. This process ensured that there was no chance that the HR managers might see their employees’ responses, and for that reason there is no risk of bias. Finally, Parts A and B were combined into a single questionnaire identified by a code for each company. Each HR manager was offered a gift worth USD 30 for their efforts.

In total, 281 questionnaires were returned (219 from EMBA students’ companies and 62 from LinkedIn respondents’ companies); of these, 256 valid responses (91.1%) were included in the data analysis. Using Harman’s single-factor test, we found that seven factors had eigenvalues that were greater than 1.0, and the first factor accounted for only 26.16% of the variance, meeting the criterion of less than 50% for no significant common method bias.

Of those sampled, 52.3% were female, and more than 90% were aged between 18 and 44 years and held a bachelor’s degree or higher. Table 1 details the respondents’ characteristics.

### 3.2. Measurement

To ensure the reliability and validity of the survey instrument, items representing the relevant constructs were developed from established scales [56,59,60,78,85,86,87,88,89,90] and were adapted for the purposes of this study. All items were measured on a five-point Likert scale (from 1 = *strongly disagree* to 5 = *strongly agree*). 

#### 3.2.1. Dependent Variable

As our focus is on employees’ overall emotional response to working from home rather than specific work issues (such as salary, promotion or colleagues), we measured EJS by using four items representing employee overall job satisfaction from Brayfield et al. [85]. This short form is reliable and has been used in previous research [91]. Sample items include the following: “I feel fairly satisfied with my present job working from home” and “I consider my job rather unpleasant”. To ensure validity, respondents were also asked “How many times have you recommended working from home for people who are used to having you around since this practice was introduced?” and “How many quarrels have you had with your colleagues since working from home was enforced?” Correlation analysis indicated that the two items were significantly correlated with respondents’ subjective evaluations (r = 0.43, *p* < 0.01 and r = −0.46, *p* < 0.01, respectively).

#### 3.2.2. Independent Variables

As WFH may in reality last for anywhere from a few days to permanently, items 1 and 2 that measure LWFH were taken from Hu et al. [56], while the remaining items were adapted from Briscese et al. [86]. We deleted two items from the LWFH scale because of low standardized factor loadings (less than 0.5). Sample items include the following: “The practice of working from home will be extended by a few months”; “The practice of working from home will be extended indefinitely for as long as is deemed necessary”.

We used one item from Nakrošienė et al. [60] to measure perceived overall HWSS and four items from Carillo et al. [59] to measure “physical” and “mental” elements of HWSS. Sample items include the following: “My home workspace is suitable for my work”; “I am bothered by noise while working at home”.

JA was measured using nine items developed by Breaugh [87]. This scale is often used in studies of job autonomy (e.g., [92]). Sample items include the following: “I am allowed to decide how to get my job done”; “I have control over how I schedule my work”; “I am allowed to modify my job objectives”.

Based on the definition of DSS during WFH, the six-item scale measuring DSS was adapted from Liang et al. [90]. This scale, which is often used in research on digital social support (e.g., [93]), incorporates two dimensions: informational support and emotional support. Sample items include the following: “When I encountered a problem, people on the digital platform would provide information to help me overcome the problem”; “When I encountered difficulties, people on the digital platform would comfort and encourage me”.

Based on the definition of MM, we adapted items 1–3 from Lautsch et al. [78], items 4–6 from Kirsch et al. [88], and items 7–9 from Kirsch [89] to measure MM on three dimensions: behaviour, output and clan control. Sample items include the following: “Our company requires employees to work the standard hours for their work group”; “Employees were evaluated by their supervisor’s observation of their results”; “Employees could negotiate with the rest of the organization when necessary”. To ensure validity, HR manager participants were also asked to send Part A of the questionnaire to their supervisors for completion. Correlation analysis confirmed that HR managers’ evaluations were significantly correlated with those of their supervisors (r = 0.64, *p* < 0.01).

#### 3.2.3. Control Variables

To reduce any variance caused by factors extraneous to the research question, we followed previous WFH studies (e.g., [34,60,94]) in controlling for employee gender, age, education level, marital status, number of children, functional specialization, organizational tenure, number of hours worked per week and experience of WFH. We used dummy variables to control for gender (1 = female, 2 = male), experience of WFH (1 = no experience, 2 = experienced) and functional specialization (1 = system analysis, 2 = marketing/sales, 3 = programming/engineering, 4 = accounting, 5 = other). Age was recorded on an interval scale using the following increments: 1 (18–24), 2 (25–34), 3 (35–44), 4 (45–54), 5 (55–65), 6 (65 and over). Education level was measured as the individual’s highest degree and was assigned to one of four groups: high school or technical secondary school, college, bachelor, master or above. Existing research also suggests that marital status and number of children may influence job satisfaction by adding to household chores [95]. For that reason, we controlled for marital status (1 = single, 2 = married or cohabiting) and number of children (0, 1, 2, 3 or more). Organizational tenure was classified in terms of five groups: 1 = less than one year; 2 = 1–2 years; 3 = 3–4 years; 4 = 5–10 years; 5 = more than 10 years. Finally, we controlled for number of hours worked per week using a four-point scale (from 1 = less than 40 h to 4 = more than 50 h).

## 4. Analysis and Results

### 4.1. Scale Evaluations

SPSS Statistics 26 (IBM, Armonk, NY, USA) and Amos 26 (IBM, Armonk, NY, USA) were used to assess reliability and validity. As shown in Table 2, the measurement model achieved goodness of fit.

Values for Cronbach’s *α* (0.830–0.920) and composite reliability (0.836–0.921) for all indicators exceeded the standard thresholds of 0.6 and 0.7 [96], respectively, indicating satisfactory reliability. Convergent validity was also confirmed, as the average variance extracted (AVE) for all constructs exceeded 0.5. Correlation coefficients for all constructs were less than the minimum square root of AVE value, indicating acceptable discriminant validity.

### 4.2. Fuzzy-Set Qualitative Comparative Analysis

As a histological method of analysis based on a multi-case comparison, fsQCA identifies common configurations in multiple cases and provides multiple equivalent paths for the same result [97]. This method was considered appropriate here to unravel the complex associations that develop between independent and dependent variables.

In fsQCA, the first step is to calibrate all measures as fuzzy sets with values ranging from 0 to 1. Using the direct calibration method for the five-point Likert scale, we set full membership threshold, crossover point and fully non-membership scores at 4, 3 and 2, respectively [97].

The second step is a necessity analysis to determine whether the presence (or absence) of a single condition is necessary for the outcome variable. Table 3 shows that the consistency of each condition was below the recommended threshold of 0.9 [98], indicating that no single factor was necessary for EJS.

In the third step of fsQCA, an algorithm produces a truth table of 2k rows (k = number of conditions), each of which represents a combination. The truth table is refined on the basis of frequency and consistency [98] (p. 44), where frequency refers to the number of observations for each combination—with a suggested threshold of 3 for samples over 150 [97]—and consistency is the extent to which cases correspond to the set-theoretic relationships expressed in a solution (which should not be less than 0.75) [98]. 

After analysing the truth table thus produced, the fsQCA software generates complex, intermediate and parsimonious solutions, and the intermediate solution output is analysed. “Core” conditions appear in both the parsimonious and intermediate solutions while “peripheral” conditions appear only in the intermediate solutions [97]. Table 4 reports three configurations for achieving high EJS, all of which have an acceptable consistency of more than 0.75.

The results identify HWSS, which appears in all three configurations, as the only core condition. Solution 1 shows that even in the case of long-term WFH and regardless of the presence or absence of JA, HWSS is assisted by DSS and MM in playing a core role in EJS enhancement. In solutions 2 and 3, JA is the peripheral condition, and LWFH is the inhibitory condition. In the presence of HWSS and JA (and the absence of LWFH), EJS can be achieved when these are combined with DSS (solution 2) or MM (solution 3).

The results of a comparative analysis based on the control variables are detailed in the Discussion section. For brevity, test results for the control variables are not provided in the text but are available on request from the corresponding author.

**Figure 2:**
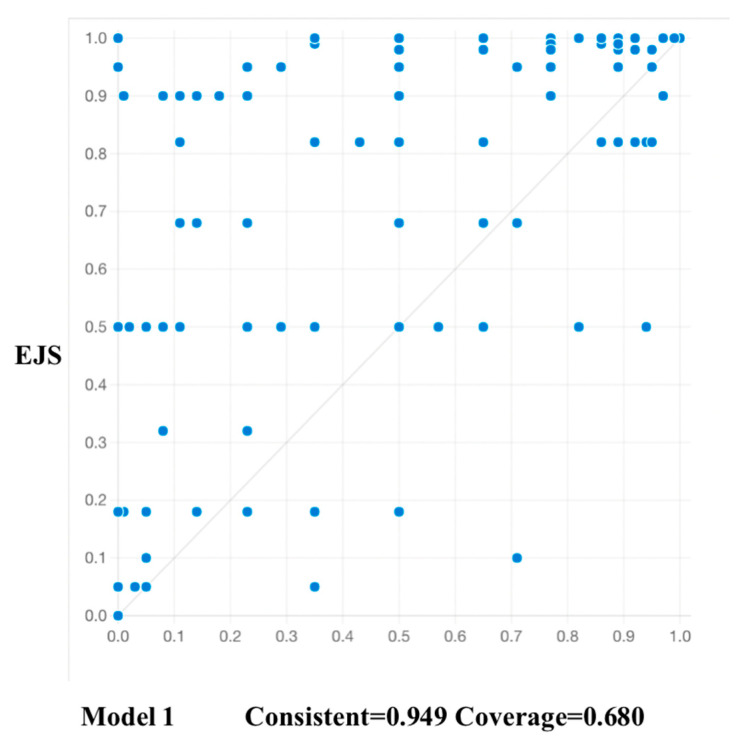
Test of Model 1 for EJS in sub-sample 1 using data from sub-sample 2.

To test predictive validity, the sample was split randomly into a modelling sub-sample (*n*_1_ = 128) and a holdout sub-sample (*n*_2_ = 128). Solutions for the modelling sub-sample from fsQCA are shown in Table 5. The model generated from this sub-sample was then tested using data from the holdout sub-sample, and Figure 2 confirms high levels of consistency and coverage (consistency > 0.75; coverage > 0.5). Predictive tests for all models confirm that the modelled sub-sample is highly predictive of the holdout sub-sample.

## 5. Discussion 

As described here, the three alternative configurations for achieving EJS represent “different routes to the same outcome”, confirming that EJS depends on configurations of job characteristics. This finding is consistent with JD-R theory, which posits that job satisfaction is the result of a combination of job demands and job resources [23] (p. 46). It also confirms the EST contention that event strength, time and space jointly determine individual emotional impacts [46].

These results also identify HWSS as a core condition for EJS. As Gray et al. [99] proposed, the workplace environment can contribute to increased job satisfaction by reducing employee depression and stress. This finding conflicts with the boundary theory argument that a weak or permeable WFH boundary between family and work will disrupt the family–work balance, with no significant impact on EJS [30]. One possible explanation is that Bellmann et al. [30] only considered minimizing the duration of WFH to offset the adverse effects of family–work conflicts on EJS while ignoring the positive effect on EJS of an appropriate combination of JA, DSS and MM.

The results also suggest that the HWSS*DSS*MM configuration can ensure EJS regardless of LWFH and JA when employees are simultaneously supported by a suitable home office, adequate digital social support and an appropriate monitoring mechanism to reduce job demands. This finding resonates with job demand–control–support theory, which posits that a combination of low demand, high support and high control can increase job satisfaction by preventing employee role overload [100].

On comparing the configurations HWSS*DSS*MM and ~LWFH*HWSS*JA*MM, it seems clear that DSS and ~LWFH*JA are interchangeable. This finding aligns with social exchange theory, which posits that a supportive work environment created by supervisors and co-workers will increase employee job autonomy, thus alleviating the pressure caused by LWFH [101].

A comparison of configurations ~LWFH*HWSS*JA*DSS and ~LWFH*HWSS*JA*MM also shows that DSS and MM have alternative effects, possibly because the performance information provided by frequent communication with work partners can reduce the need for feedback during the supervision process [76], and interaction with supervisors can reduce the loneliness caused by home isolation [102]. At the same time, HWSS*JA*DSS or HWSS*JA*MM can achieve high EJS during short-term WFH. This finding conflicts with Carillo et al.’s [59] argument that employees will become more acculturated and satisfied the longer that WFH lasts. One possible explanation is that they may be ignoring the adverse effects of social isolation during prolonged WFH, which offsets the impact of event strength.

Beyond the above, our findings suggest that JA is not a prerequisite for EJS. This challenges the traditional assumption that job autonomy—as a core job characteristic—is more likely to alleviate emotional exhaustion and promote positive attitudes [103]. One possible explanation is that COVID-19 exacerbates the hazards of WFH, such as social isolation, family–work conflict, role overload, after-hours work-related technology use and stress, which cannot be completely offset by JA alone [104]. A second possibility is that long-term WFH reduces or eliminates the constant supervision and interpersonal interaction with colleagues or supervisors associated with working in the office, and as such the importance of job autonomy was weakened in the minds of home workers. This means that EJS also depends on the synergy between ~LWFH, JA, HWSS and DSS (or MM).

Finally, the comparative analysis of control variables yielded a number of interesting results: (1) Female employees place more emphasis than males on DSS, possibly because social support serves to mitigate the adverse effects of pressure on women’s well-being [105]. (2) MM only inhibits EJS among employees aged 35–44, perhaps because family needs are a more significant issue for this age group and more autonomy is needed, as too much supervision may cause work–family conflicts and increase dissatisfaction [95]. (3) LWFH has a greater inhibitory effect on EJS among more highly educated employees, while HWSS for them is less important. This may reflect their pursuit of more spiritual satisfaction beyond their basic needs, as well as social needs that cannot be met by long-term WFH [58]. (4) The more children an employee has, the more important HWSS and JA become. One possible explanation is that employees may struggle with work and home boundary violations due to the collocation of work and home, increasing the number of unfinished tasks in both domains and decreasing satisfaction with both domains. Thus, an independent workspace enables employees to create a physical boundary between work and private life, thereby preventing blurring and conflicts between family–work boundaries [36]. For employees who need to look after their children, additional autonomy reflects supervisors’ care and trust, and employees are likely to be more engaged with their work [14]. (5) DSS is a limiting condition for married employees, perhaps because married employees receive social support from their partners (and children) outside of work, and too much social support may lead to information overload [106]. (6) DSS can also have an inhibiting effect, and MM is more important to employees with WFH experience. One possible explanation is that because they do not need extra social support to adapt to WFH [107], they are more concerned about increased workloads and supervisory neglect because their colleagues are unused to WFH. (7) LWFH is only a problem for employees who work less than 40 h per week and whose organizational tenure is less than 2 years or more than 10 years. This may be because they are more sensitive to issues of belonging, which may be affected by long-term WFH. (8) HWSS is a core condition for WFH marketing and sales employees; because digital tools are their only means of communicating with customers, a quiet workspace and access to essential IT tools determine their productivity [108].

## 6. Conclusions

Drawing on event system theory, this study employed fsQCA to explore how EJS is affected by the alternative configurations of five antecedents of WFH event strength, time and space. The findings indicate that three configurations promote EJS. While HWSS is a core condition for EJS, LWFH has an inhibitory effect unless HWSS, DSS and MM are appropriately combined. JA is not a necessary condition for high EJS, and the longer WFH lasts, the less important JA is to employees. These findings have a number of clear implications for theory and practice.

### 6.1. Theoretical Implications

1. The study extends the WFH literature beyond voluntary contexts, given that few studies have investigated enforced WFH [41]. By analysing how different configurations of job characteristics have affected EJS during enforced WFH throughout the COVID-19 pandemic, the study’s findings enrich the WFH literature and offer new insights for the design of future hybrid office models.
2. This study enriches the literature on WFH supervision strategy management by introducing the concept of MM. According to Wang et al. [38], appropriate monitoring can alleviate employee procrastination and enhance job satisfaction. In contrast, boundary and control theories contend that monitoring prevents employees from fulfilling family responsibilities, with adverse effects on well-being [109]. The present findings enrich the literature on monitoring mechanisms for enforced WFH by conceptualizing MM in terms of behaviour, output and clan control and exploring how MM interacts with other job characteristics to promote EJS. Our findings indicate that EJS during long-term WFH depends on the synergy between MM, HWSS and DSS. This new perspective illuminates the “black box” of MM’s impact on EJS in contexts where job autonomy becomes less important to employees, as in the case of working during the COVID-19 pandemic.
3. The study augments the WFH literature on job characteristics by exploring antecedent configurations of job characteristics that promote EJS during enforced WFH. Unlike previous approaches that have focused on a single job characteristic, the present study draws on EST to deconstruct WFH characteristics along the dimensions of event strength, space and time. Using fsQCA, the study identifies the conditional combinations of job characteristics that promote EJS and responds to Rymaniak et al.’s [110] call for more research into the optimal implementation of WFH.
4. While EST is typically applied to reactive events, many other events can be strategically framed in this way to produce desired outcomes [46]. The present study demonstrates an important extension of EST by treating WFH as proactive event.

### 6.2. Managerial Implications

This study also identifies a number of ways in which managers can enhance EJS during the COVID-19 pandemic.

1. As HWSS is a core condition for achieving EJS, enterprises should instruct their employees to ensure that they maintain an undisturbed work environment by consciously avoiding family distractions, creating an independent workspace and keeping family members informed about their work schedule. For employees who lack the necessary resources, enterprises should provide assistance, including financial subsidies for essential office equipment.
2. When implementing long-term enforced WFH, enterprises should ensure that MM, HWSS and DSS function together optimally as the basis for high EJS.
3. During short-term WFH, ensuring HWSS and JA allows DSS and MM to be interchangeable. Enterprises with inadequate DSS can therefore supervise employees through multiple channels, using performance feedback and timely communication to reduce information uncertainty.
4. The configurations ~LWFH*JA*DSS*~MM or ~LWFH*JA*~DSS*MM can help married employees to achieve EJS. This suggests that enterprises should avoid the simultaneous strengthening of DSS and MM for married employees when short-term WFH supports JA. Enterprises can improve married employees’ job satisfaction by adjusting the frequency of supervision in a timely fashion or by utilizing virtual technologies, such as artificial reality, to enhance interactivity. For employees who work less than 40 h per week or whose organizational tenure is less than 2 years or more than 10 years, LWFH tends to inhibit EJS. Enterprises should prioritize these workers for hybrid office arrangements and psychological support that mitigate the adverse effects of long-term WFH on physical and mental health. As employees with two children emphasize the importance of JA, enterprises should provide support in the form of (1) time management skills to help employees balance child-care and work and (2) online training in self-leadership to cultivate work engagement and autonomy. Finally, enterprises should take steps to improve EJS on the basis of individual characteristics. They should, for example, provide more DSS for female employees, reduce MM for employees aged 35–44, reduce LWFH for highly educated employees, reduce DSS and increase MM for employees with WFH experience and improve HWSS for marketing and sales employees.

### 6.3. Limitations and Future Research Directions

The limitations of the present study serve to highlight valuable directions for future research.

1. These proposals for the rational design of a hybrid model combining office working and WFH that can effectively predict organizational and employee-level outcomes, such as the impact of workload on job satisfaction, invite further research in a post-pandemic era.
2. While the study focuses on the impact of WFH at employee level, managers’ attitudes to WFH are equally important because they decide whether to implement WFH. Rose et al. [111] found that the long-term implementation of enforced WFH during the COVID-19 pandemic can change hostile managerial attitudes to WFH. Future research should investigate which WFH job characteristics affect the attitudes and behaviours of middle and senior managers.
3. The use of cross-sectional data to explore the combined effects of WFH job characteristics in terms of event time, space and strength invites further investigation of the varying impacts of WFH event strength on EJS in relation to spatial and temporal change.
4. Future studies should employ other measures aside from self-reporting to invite managers to assess employees’ JA. Other approaches might include asking family members to evaluate employees’ HWSS, using wearable devices, such as electronic watches, to measure noise when WFH in order to eliminate common method bias.
5. While this study has controlled for a range of employee characteristics, future research on the relationship between WFH and EJS should take account of personality characteristics and individual preferences that were beyond the scope of the present article.
6. This fsQCA-based exploration of antecedent configurations for optimizing EJS should be supplemented by other qualitative methods (e.g., interview, observation and field experience) to disclose the inherent causal logic of each configuration.

## Figures and Tables

**Table 1:** The descriptive statistics of respondents’ characteristics.

Items	Frequency Counts	Percentage (%)
**Gender**		
Female	134	52.3
Male	122	47.7
**Age**		
18–24	85	33.2
25–34	90	35.1
35–44	57	22.3
45–54	23	9.0
55–65	1	0.4
**Education level**		
High school or technical secondary school	7	2.7
College	16	6.3
Bachelor	131	51.2
Master or above degree	102	39.8
**Marital status**		
Single	131	51.2
Marriage or cohabitation	125	48.8
**Number of children**		
0	154	60.2
1	76	29.7
2	26	10.1
**Organizational tenure (years)**		
Less than 1	90	35.2
1–2	48	18.8
3–4	31	12.1
5–10	31	12.1
More than 10	56	21.8
**Functional specialization**		
System analysis	14	5.5
Marketing/sales	76	29.7
Programming/engineering	30	11.7
Accounting	15	5.8
Other	121	47.3
**Number of hours worked per week**		
Less than 40	155	60.5
40–45	57	22.3
46–50	21	8.2
More than 50	23	9.0
**Experience of WFH**		
No experience	137	53.5
Experienced	119	46.5

**Table 2:** Means, standard deviations, and assessment of convergent and discriminant validity of reflective constructs.

Variables	1	2	3	4	5	6	7	8	9	10	11	12	13	14	15
1. Gender	-														
2. Age	0.19 **	-													
3. Education level	0.13 *	0.05	-												
4. Marital status	0.13 *	0.71 **	−0.01	-											
5. Number of children	0.16 **	0.67 **	−0.01	0.71 **	-										
6. Organizational tenure	0.21 **	0.76 **	−0.03	0.67 **	0.63 **	-									
7. Functional specialization	−0.09	0.05	0.00	−0.01	−0.03	−0.02	-								
8. Number of hours worked per week	0.00	0.07	−0.09	0.11	0.07	0.05	−0.13 *	-							
9. Experience of WFH	0.02	0.18 **	0.14 *	0.20 **	0.12	0.13 *	0.03	0.11	-						
10. LWFH	−0.12 *	−0.04	−0.13 *	−0.08	0.04	−0.12 *	0.01	0.12	−0.03	**0.81**					
11. HWSS	−0.07	−0.01	0.05	0.07	0.08	−0.05	−0.06	0.03	0.15 *	0.27 **	**0.72**				
12. JA	−0.06	−0.06	0.00	−0.05	−0.09	0.01	−0.05	0.12	0.11	−0.03	−0.03	**0.71**			
13. DSS	−0.06	0.01	−0.01	0.07	0.10	−0.09	−0.02	0.04	0.09	0.30 **	0.43 **	−0.05	**0.81**		
14. MM	−0.03	−0.11	−0.01	−0.18 **	−0.07	−0.19 *	−0.08	0.11	0.12	0.23 *	0.31 **	−0.12	0.40 **	**0.72**	
15. EJS	0.05	0.02	0.05	0.07	0.10	−0.01	−0.02	−0.02	0.17 **	0.27 **	0.56 **	−0.09	0.54 **	0.43 **	**0.80**
**Mean**	1.48	2.08	3.28	1.49	1.50	2.67	3.60	1.66	1.47	2.40	3.36	3.64	3.52	3.47	3.53
**Standard deviation**	0.50	0.97	0.70	0.50	0.67	1.58	1.46	0.97	0.50	1.06	0.87	0.66	0.82	0.71	0.77
**Cronbach’s alpha**	-	-	-	-	-	-	-	-	-	0.83	0.83	0.90	0.92	0.90	0.88
**Composite reliability**	-	-	-	-	-	-	-	-	-	0.85	0.84	0.90	0.92	0.90	0.88
**AVE**	-	-	-	-	-	-	-	-	-	0.66	0.51	0.51	0.66	0.52	0.64

Note: Diagonal elements (in bold) are the square root of the AVE. Off-diagonal elements are the correlations among constructs; * *p* < 0.05, ** *p* < 0.01 (two-tailed). Abbreviations: EJS denotes employee job satisfaction; LWFH denotes longevity of WFH; HWSS denotes home workspace suitability; JA denotes job autonomy; DSS denotes digital social support; MM denotes monitoring mechanism; AVE denotes average variance extracted.

**Table 3:** Analysis of necessary conditions.

Causal Conditions	Consistency	Coverage
LWFH	0.402149	0.901668
~LWFH	0.700496	0.726307
HWSS	0.813940	0.887055
~HWSS	0.342534	0.694947
JA	0.826006	0.752270
~JA	0.276639	0.885382
DSS	0.866887	0.874208
~DSS	0.319394	0.762562
MM	0.856033	0.847534
~MM	0.327768	0.818519

Abbreviations: LWFH denotes longevity of WFH; HWSS denotes home workspace suitability; JA denotes job autonomy; DSS denotes digital social support; MM denotes monitoring mechanism.

**Table 4:** Configurations for achieving high levels of EJS.

Configuration	Solution		
	1	2	3
LWFH		⊗	⊗
HWSS	⬤	⬤	⬤
JA		●	●
DSS	●	●	
MM	●		●
Consistency	0.957	0.954	0.939
Raw coverage	0.679	0.441	0.432
Unique coverage	0.281	0.043	0.034
Solution consistency	0.943		
Solution coverage	0.755		

Note: Black circles (●) indicate the presence of a condition, and circles with “×” (⊗) indicate its absence. Large circles indicate core conditions, small ones indicate peripheral conditions. Abbreviations: EJS denotes employee job satisfaction; LWFH denotes longevity of WFH; HWSS denotes home workspace suitability; JA denotes job autonomy; DSS denotes digital social support; MM denotes monitoring mechanism.

**Table 5:** Solutions for high EJS for sub-sample 1.

EJS			
Configurations	Raw Coverage	Unique Coverage	Consistency
1.HWSS*DSS*MM	0.678	0.325	0.966
2.~LWFH*HWSS*JA*DSS	0.401	0.048	0.967
3.~LWFH*HWSS*JA*MM	0.380	0.027	0.962
Solution consistency: 0.958			
Solution coverage: 0.753			

Abbreviations: EJS denotes employee job satisfaction; LWFH denotes longevity of WFH; HWSS denotes home workspace suitability; JA denotes job autonomy; DSS denotes digital social support; MM denotes monitoring mechanism.

## Data Availability

The data presented in this study are available on request from the corresponding author. The data are not publicly available due to privacy or ethical restrictions.

## Appendix A

**Table A1:** Part A of the questionnaire on working from home arrangements and job satisfaction during the COVID-19 pandemic.

Category (Number of Questions)	Example of Questions	Answer Options
Monitoring Mechanisms (9)	Our company requires employees to work the standard hours for their work group.	1 = “strongly disagree”, to 5 = “strongly agree”
	Supervisors contacted with employees frequently every day.	
	Our company requires employees to separate work and family.	
	Employees were evaluated by their supervisor’s observation of their results.	
	Supervisors placed significant weight upon timely project completion.	
	Supervisors used pre-established targets as benchmarks for employees’ performance evaluations.	
	Employees actively participated in project meetings to understand the project’s goals, values, and norms.	
	Employees were encouraged to adopt those behaviours that fit our company’s values and norms.	
	Employees could negotiate with the rest of the organization when necessary.	

**Table A2:** Part B of the questionnaire on working from home arrangements and job satisfaction during the COVID-19 pandemic.

Category (Number of Questions)	Example of Questions	Answer Options
Socio-demographic characteristics	What is your gender?	Female, Male
	How old are you?	18–24, 25–24, 35–44, 45–54, 55–65, 65 and over
	What is your education level?	High school or technical secondary school, College, Bachelor, Master or above degree
	What is your marital status?	Single, Marriage or cohabitation
	How many children do you have?	0, 1, 2, 3 or above
	What is your organizational tenure (years)?	Less than 1, 1–2, 3–4, 5–10, More than 10
	What is your functional specialization?	System analysis, Marketing/sales, Programming/engineering, Accounting, Other
	How many hours did you work per week during working from home?	Less than 40, 40–45, 46–50, More than 50
	Have you experienced working from home before COVID-19?	No, Yes
Longevity of working from home (5)	The practice of working from home will only last for a few days.	1 = “strongly disagree”, to 5 = “strongly agree”
	The practice of working from home will be extended by a few weeks.	
	The practice of working from home will be extended by a few months.	
	The practice of working from home will be extended by a year.	
	The practice of working from home will be extended indefinitely for as long as is deemed necessary.	
Home workspace suitability (5)	My home workspace is suitable for my work.	1 = “strongly disagree”, to 5 = “strongly agree”
	I am not easy to get distracted working at home.	
	I am bothered by noise while working at home	
	I have good conditions to work from home.	
	I have satisfactory access to professional IT tools from home (professional software, messaging, shared files, video conference …).	
Job autonomy (9)	I am allowed to decide how to get my job done.	1 = “strongly disagree”, to 5 = “strongly agree”
	I am allowed to choose the way to go about my job (the procedures to utilize).	
	I am allowed to choose the methods to use in carrying out my work.	
	I have control over how I schedule my work.	
	I have control over the sequencing of my work activities (when I do what).	
	I am allowed to decide when to do particular work activities.	
	I am allowed to modify the normal way we are evaluated so that I can emphasize some aspects of my job and play down others.	
	I am allowed to modify my job objectives to accomplish.	
	I have control over what I am supposed to accomplish.	
Digital social support (6)	When I needed help, people on the digital platform would offer suggestions to me.	1 = “strongly disagree”, to 5 = “strongly agree”
	When I encountered a problem, people on the digital platform would provide information to help me overcome the problem.	
	When I encountered difficulties, people on the digital platform would help me discover the cause and provide me with suggestions.	
	When I encountered difficulties, people on the digital platform would accompany me through the difficulties.	
	When I encountered difficulties, people on the digital platform would comfort and encourage me.	
	When I encountered difficulties, people on the digital platform would listen to me talk about my private feelings.	
Employee job satisfaction (4)	Most days I was enthusiastic about my work when I work from home.	1 = “strongly disagree”, to 5 = “strongly agree”
	I feel fairly satisfied with my present job working from home.	
	I find real enjoyment in my work.	
	I consider my job rather unpleasant.	
